# Hospital organizational context and delivery of evidence-based stroke care: a cross-sectional study

**DOI:** 10.1186/s13012-018-0849-z

**Published:** 2019-01-18

**Authors:** Nadine E. Andrew, Sandy Middleton, Rohan Grimley, Craig S. Anderson, Geoffrey A. Donnan, Natasha A. Lannin, Enna Striol-Salama, Brenda Grabsch, Monique F. Kilkenny, Janet E. Squires, Dominique A. Cadilhac, On behalf of the Stroke123 Investigators

**Affiliations:** 1 Stroke and Ageing Research, Department of Medicine, School of Clinical Sciences at Monash Health, Monash University, Level 3, Hudson Institute Building, 27-31 Wright Street, Clayton, VIC 3168 Australia; 2 Department of Medicine, Peninsula Clinical School, Central Clinical School, Monash University, Melbourne, Australia; 3 Nursing Research Institute, St Vincent’s Health Australia (Sydney) and Australian Catholic University, Sydney, Australia; 4 Sunshine Coast Clinical School, The University of Queensland, Birtinya and Statewide Stroke Clinical Network, Queensland Health, Brisbane, Australia; 5 The George Institute for Global Health, Faculty of Medicine, University of New South Wales, Sydney, Australia; 6 Florey Institute of Neuroscience and Mental Health, University of Melbourne, Heidelberg, Australia; 7 Faculty of Health Sciences, La Trobe University, Bundoora, Australia; 8 Occupational Therapy Department, Alfred Health, Melbourne, Australia; 9 Australian Bronchiectasis Registry, Lung Foundation Australia, Brisbane, Australia; 10 School of Nursing, Faculty of Health Sciences, University of Ottawa, Ottawa, Ontario Canada

**Keywords:** Organizational context, Evidence-based care, Stroke, Stroke unit

## Abstract

**Background:**

Organizational context is one factor influencing the translation of evidence into practice, but data pertaining to patients with acute stroke are limited. We aimed to determine the associations of organizational context in relation to four important evidence-based stroke care processes.

**Methods:**

This was a mixed methods cross-sectional study. Among 19 hospitals in Queensland, Australia, a survey was conducted of the perceptions of stroke clinicians about their work using the Alberta Context Tool (ACT), a validated measure covering 10 concepts of organizational context, and with additional stroke-specific contextual questions. These data were linked to the Australian Stroke Clinical Registry (AuSCR) to determine the relationship with receipt of evidence-based acute stroke care (acute stroke unit admission, use of thrombolysis for those with acute ischemic stroke, receipt of a written care plan on discharge, and prescription of antihypertensive medications on discharge) using quantile regression. Exploratory cluster analysis was used to categorize hospitals into high and low context groups based on all of the 10 ACT concepts. Differences in adherence to care processes between the two groups were examined.

**Results:**

A total of 215 clinicians completed the survey (50% nurses, 37% allied health staff, 10% medical practitioners), with 81% being in their current role for at least 1 year. There was good reliability (∞ 0.83) within the cohort to allow pooling of professional groups. Greater ACT scores, especially for social capital (μ 9.00, 95% confidence interval [CI] 4.86 to 13.14) and culture (μ 7.33, 95% CI 2.05 to 12.62), were associated with more patients receiving stroke unit care. There was no correlation between ACT concepts and other care processes. Working within higher compared to lower context environments was associated with greater proportions of patients receiving stroke unit care (88.5% vs. 69.0%) and being prescribed antihypertensive medication at discharge (62.5% vs. 52.0%). Staff from higher context hospitals were more likely to value medical and/or nursing leadership and stroke care protocols.

**Conclusions:**

Overall organizational context, and in particular aspects of culture and social capital, are associated with the delivery of some components of evidence-based stroke care, offering insights into potential pathways for improving the implementation of proven therapies.

**Electronic supplementary material:**

The online version of this article (10.1186/s13012-018-0849-z) contains supplementary material, which is available to authorized users.

## Introduction

Stroke is a leading cause of premature death and disability worldwide [1]. Despite the widespread availability of systematic reviews, clinical guidelines, and national performance evaluations to support high-quality stroke care [2–4], evidence-practice gaps deny many patients from receiving recommended care [5]. For example, a recent national audit of stroke care in Australia found that only 67% of hospitalized patients received stroke unit care (i.e., care in an organizational unit characterized by co-located beds within a geographically defined unit staffed by a multidisciplinary team with a special interest and training in stroke and/or rehabilitation), and only 13% of those with acute ischemic stroke (AIS) received thrombolytic treatment with intravenous recombinant tissue plasminogen activator (rtPA) [3], both of which have been shown to improve outcomes [6, 7]. Explanations for variations in practice are complex, and research on the factors influencing the implementation of evidence are limited.

Although no clear definition of context exists [8], we defined organizational context in relation to healthcare according to Rycroft-Malone 2004 with reference to the Promoting Action on Research Implementation in Health Services (PARIHS) framework [9], that is the internal or work environments in which healthcare is delivered. This is distinct from outer context, that is factors beyond the healthcare organization such as social systems, policy, and legislation [8–12], and from individual features such as the skill level and role of staff [8]. Organizational context is considered crucial not only to effective knowledge translation (i.e., the synthesis, dissemination, and application of knowledge) but also to the sustainability and generalisability of knowledge translation activities. Consequently, it is an important component of several implementation frameworks, such as the PARIHS [9], Theoretical Domains Framework [10, 13], and the Consolidated Framework for Implementation Research [14].

Given the influence of barriers at organizational and multidisciplinary team levels, a greater understanding of the relationship between knowledge translation and organizational context is important for improving the implementation of synthesized knowledge such as guideline-recommended care [15]. Since 2007, the Stroke Foundation of Australia has performed a national audit to measure adherence to the national clinical guidelines for stroke and to promote improvements to evidence-based stroke care by providing performance reports back to hospitals. In Queensland, feedback has also been facilitated using workshops at individual hospitals and combined clinical fora in which results are discussed for all hospitals and local evidence to practice gaps highlighted for action. However, information on how specific contextual factors influence the application of this knowledge is limited.

The aim of this study was to determine the relationship between various aspects of the organizational context of hospitals and effectiveness of knowledge translation, measured by adherence to the delivery of four evidence-based, nationally endorsed processes of care for patients with acute stroke. These were stroke unit care, thrombolysis treatment for AIS, discharge care planning, and prescription of antihypertensive medications at discharge. Our hypothesis was that a more positive organizational context would be associated with better knowledge translation as indicated by greater adherence to evidence-based stroke care.

## Methods

### Design

This was a mixed methods cross-sectional study. Survey data obtained from staff (doctors, nurses, allied health, and managers) at 19 acute care hospitals in Queensland, Australia, between November 2013 and September 2014 were linked to patient-level data of the national, prospective Australian Stroke Clinical Registry (AuSCR). These data were collected during the pre-intervention (baseline) phase of a large, quality improvement intervention study (Stroke123) [16]. The study was approved by the Metro South Human Research Ethics Committee and Monash University, and each participating hospital provided site governance authorization.

### Description of the acute care hospitals

Hospitals included both metropolitan (*n* = 12) and regional (*n* = 7) sites comprising 19 of 23 hospitals with stroke units in Queensland. There were few differences between the metropolitan and regional sites in terms of annual stroke admissions and access to specialist resources. All of the regional and all but 2 of the metropolitan hospitals offered thrombolysis, and all had a stroke unit. They all provided multidisciplinary care and regularly participated in local and national stroke audit programs and Queensland Statewide Stroke Clinical Network biannual fora. Most (74%) provided data on over 100 patients with stroke in AuSCR during 2013.

### Participant recruitment and selection

Staff who worked predominantly on the stroke unit were eligible to participate. Allied health staff were qualified and registered health professionals as required by hospital standards in Australia and included physiotherapists, occupational therapists, speech pathologists, social workers, and dieticians.

### Instrument

The staff survey had two components and included closed and open questions. Part 1 involved the use of a project-specific self-completed questionnaire designed by the investigators and was based on previous evaluation research of stroke services by DAC. These questions covered the respondent’s characteristics (*N* = 7 items including age, sex, time since qualification, current professional position, employment status, and education level). It also contained 20 stroke-specific questions pertaining to the provision of evidence-based acute stroke care. Responses to the first 17 were graded on a 5-point Likert scale (strongly disagree to strongly agree), covering management involvement, teamwork and staff, readiness for organizational change, and the broader work environment. In question 18, participants were asked about the types of performance data they used and to rate the usefulness of these from 1 to 10. In question 19, they were asked about prior quality improvement experience. Question 20 contained 3 open-ended questions related to the potential barriers and enablers in the delivery of stroke care. The survey can be found in Additional file 1.

Part 2 of the survey involved the hospital staff completing the Alberta Context Tool (ACT) to measure organizational context [17, 18]. We invited hospital staff to complete the version of the ACT matched to their professional group with reference to the stroke unit that they worked in most of the time (since some of the health professionals worked in different locations within the same hospital or between hospitals).

The ACT (Copyright, Estabrooks 2007) [17, 18] was designed to assess organizational context within complex healthcare settings with a focus on potentially modifiable concepts [17, 19, 20] outlined in Table 1. The ACT comprises 10 individual concepts across 8 dimensions: leadership, culture, evaluation, social capital, informal interactions, formal interactions, structural/electronic resources, and organizational slack (staff, space, and time). Seven of these concepts use scale-based scoring (5-point Likert scale from “strongly agree” to “strongly disagree”) and 3 (formal interactions, informal interactions, and structural/electronic resources) are count-based (i.e., list of activities). The ACT is based on the PARIHS framework incorporating concepts of leadership, culture, and evaluation [9] as well as a large synthesis of work on the determinants of adoption of scientific knowledge translation [21, 22]. Of the different versions of the tool that have been developed for different health care settings and professional groups [17], the acute care version was used for this project. Versions specific to nursing, allied health, medical, and management were provided. These allowed for between 56 and 58 questions depending on the respondents’ profession.

**Table 1:** Alberta Context Tool concepts

Concept	Definition
1. Leadership*	Actions of formal leaders to influence change and excellence in practice
2. Culture*	Reflects a supportive work culture
3. Evaluation*	Using data to assess team performance and achieve outcomes
4. Social capital	Active connections among people
5. Informal interactions	Information exchanges that promote transfer of knowledge
6. Formal interactions	Scheduled activities that promote transfer of knowledge
7. Structural/electronic resources	Elements that facilitate the ability to assess and use knowledge
Organisational slack	The cushioning of resources that allows an organization to adapt to pressures for changes
8. Staff	
9. Space	
10. Time	

Estabrooks et al. [17]

*Primary components of the PARIHS context domain

### Data collection

The Stroke123 intervention involved an externally facilitated workshop to review clinical performance data and develop an action plan to address local barriers to providing evidence-based care [23]. Staff were provided an opportunity to complete the surveys prior to the workshop and were given an additional opportunity to complete a copy on the day. Survey data were collected anonymously.

Routinely collected data from each participating hospital in AuSCR between 1 January and 31 December 2013 were used to calculate the proportion of patients receiving the four quality of care processes, selected on the basis of being established as a quality indicator through a consultative process [24]. AuSCR is an ongoing, prospective, national clinical registry designed to monitor the quality of acute care provided to hospitalized patients with acute stroke or transient ischemic attack (TIA) [16, 24]. All hospitals participating in the Stroke123 study were required to contribute data prospectively to AuSCR. Eligibility for the four quality of care processes were stroke unit care (all patients), thrombolysis (AIS patients), discharge care plan outlining management (for patients discharged directly to the community from acute care), and prescribed antihypertensive medication(s) at discharge (all patients).

### Statistical analysis

Responses from the staff survey were dichotomized as positive (yes) for coding as “agree” or “strongly agree” and negative (no) if the response was “strongly disagree”, “disagree,” or “neutral.” Scoring of responses to the ACT was according to the developer guidelines [18], with the score for each concept calculated from the total scores divided by the total number of items in each concept. An average unit score was also calculated for each concept by calculating the average score of the clinicians for that unit. Where data were missing for an item within a concept, the overall concept was calculated based on the average of recorded items in that section.

Comparisons were made to determine if ACT scores could be combined across professions. Since there were no significant differences in mean concept scores between disciplines, the main analyses used data combined for the different professional groups. However, responses from medical and allied health staff (who often worked across units) were also reported separately to those from nursing staff (who often work solely within units). To achieve this, we needed to recode the informal interaction concept as it contained different response options for different professions. This meant that items in this concept that were not common across all versions were excluded so that the total score for all respondents for that dimension (regardless of discipline) was out of 7 rather than a range of 7 to 10.

Text responses to open-ended questions were examined using inductive content analysis by an author (NA) by coding the transcribed text into themes. Major themes for each hospital were identified and grouped into three main components and discussed with a second author (DC). Any uncertainty in coding was discussed with the two authors until consensus was reached. Themes were also examined according to whether they were from low or high context groups.

Descriptive statistics were used according to the distribution and nature of the data, to compare patient and clinician demographics between units and professions.

Intraclass correlation (ICC) was calculated using one-way analysis of variance (ANOVA) to provide a measure of agreement about the group mean within stroke units. The hypothesis was that the level of aggregation at the unit level was not different between nursing and allied health/medical staff, tested by calculating the group-specific ICC. In psychological research, values > 0.1 indicate that data are suitable for aggregation at a higher level [17, 19]. Confirmatory factor analysis (CFA) was used to determine the internal validity of the ACT concepts and to assess whether the observable variables adequately represented the latent constructs of the sample. The results obtained from the CFA identified poorly fitted items in the concepts of culture, evaluation, resources, and time. These items were trimmed (i.e., poorly fitted items removed) and analyses re-run to determine the impact on the overall results. As this impact was minimal, the main results are reported using all ACT items to maximize external validity [25].

Data were aggregated at a unit level for sites that contained eight or more responses (*n* = 14), and exploratory cluster analyses were applied to find groups within the data based on site scores for each of the ten ACT concepts. Hierarchical cluster analyses were performed to determine the most appropriate number of clusters. *K*-means clustering was then applied based on the similarities in means across the ten ACT concepts, with the number of clusters pre-specified according to the hierarchical cluster results [26]. Differences in mean scores for each ACT concept were assessed between the clusters to ensure they were sufficiently distinct. As there were two naturally occurring groupings with consistently lower or higher means scores for each of the concepts, these two clusters were defined as high or low context groups.

Adherence to each care process was calculated from the number of patients who were eligible for that care process. Quantile regression was used to determine associations between the ACT concept scores and adherence to each of the four care processes. This was performed for the group as a whole and also stratified according to each staff professional group. Between-group differences in adherence to the four care processes were derived from cluster analysis, and comparisons between units were grouped according to the higher or lower context from cluster analysis. A standard significance level of *p* < 0.05 was used. All analyses were performed using Stata IC version 12.

## Results

A total of 215 responses, median 11 (quartile 1, quartile 3: 6, 16) to the survey were received from the 19 participating hospitals. A response rate could not be determined as the total number of eligible participants was unknown since some staff worked across multiple units and surveys were distributed using multiple methods making accurate tracking difficult. Most respondents were nurses (50%), followed by allied health (37%) and medical doctors (10%). Approximately one quarter was aged less than 30 years, 78% were female, 81% had been in their current role for one or more years, and 12% had a masters or PhD in addition to their bachelor degree. There was significant variation between the units according to the participating professions, times in current roles, and level of education level, but not with regard to age or sex (Additional file 2: Table S1).

Variation existed between hospitals in relation to perceptions of overall support, two-way communication, and clear communication across all levels of an organization. For example, the proportion of staff that “agreed” or “strongly agreed” that executive staff provided adequate support, and two-way communication was 57% overall (range 23 to 80%) and 41% overall (range 0 to 70%), respectively. Overall, 72% (range 17 to 100%) of staff felt their unit had a stable workforce most of the time, and 78% reported familiarity with stroke protocols and policies. However, at two sites, less than 30% of staff reported being familiar with these protocols (Table 2). Results stratified by high and low context groups are also reported in Table 2.

**Table 2:** Project-specific survey results according to overall context categories

	All (%), *N* = 212	Low context* (%), *N* = 51	High context (%), *N* = 134	*p* value
Management involvement				
Adequate executive support	56.7	43.1	61.5	0.03
Good communication between management and staff	40.9	33.0	45.32	0.1
Regular updates provided by management	45.2	43.1	48.5	0.5
Teamwork and staff				
Effective multidisciplinary communication	85.2	80.4	90.2	0.07
Stable workforce	72.1	70.6	77.1	0.4
Staff are familiar with stroke protocols	78.1	66.7	84.9	0.006
Medical staff engagement that facilitates stroke care	70.7	68.0	76.5	0.2
Allied health staff engagement that facilitates stroke care	92.2	88.0	95.4	0.08
Nursing staff engagement that facilitates stroke care	79.3	62.7	86.4	< 0.001
Organizational change				
Sufficient opportunities to question management about change	61.2	52.9	62.9	0.2
Staff are always consulted about change	37.2	34.0	38.2	0.6
Changes are communicated clearly	35.9	31.4	37.4	0.5
The introduction of stroke protocols has been effective	82.8	68.6	89.4	0.001
Patients and families have access to adequate information	60.4	46.9	65.9	0.02
Other				
The QSSCN had a positive impact on stroke care at a hospital level	72.7	64.7	75.8	0.1
The QSSCN had a positive impact on stroke care at a Health Network level	70.7	66.0	72.5	0.4
Professional development has improved knowledge about stroke care	65.4	64.7	65.7	0.9
Hospital performance data has been used in the last 12 months	65.8	59.4	67.5	0.8
Participated in quality improvement activities to improve stroke care	61.0	58.0	61.6	0.7

*QSSCN* Queensland Statewide Stroke Clinical Network

*Overall context grouping was determined using exploratory cluster analysis to categorize units into two clusters based on responses to the Alberta Context Tool moderate context (*N* = 4 sites) and high context (*N* = 10 sites). Excludes responses from staff at sites with < 8 completed surveys (*N* = 5 sites, 27 responses)

Multidisciplinary communication was generally strong across all sites, with 85% of respondents reporting that it facilitated effective care, and only 5% reported it being inadequate. The role of allied health was well valued, with 92% of respondents reporting that allied health staff engagement facilitated better stroke care. At all sites, staff reported that the introduction of protocols and care pathways had been effective, and near two thirds felt that the Queensland Statewide Stroke Clinical Network had positively influenced practice (Table 2).

Overall, 93% of the ACT data were complete; missing data from individual concepts ranged from 2 to 9%. Cronbach’s alpha for all ten ACT concepts showed high internal consistency (∞ range 0.80 to 0.82, overall 0.83). Internal consistency was consistent across professional groups (ranges: nursing 0.78–0.81, allied health/medical 0.81–0.84). Reliability was also within acceptable levels for item-rest and inter-item correlations (Additional file 3: Table S2).

**Fig. 1:**
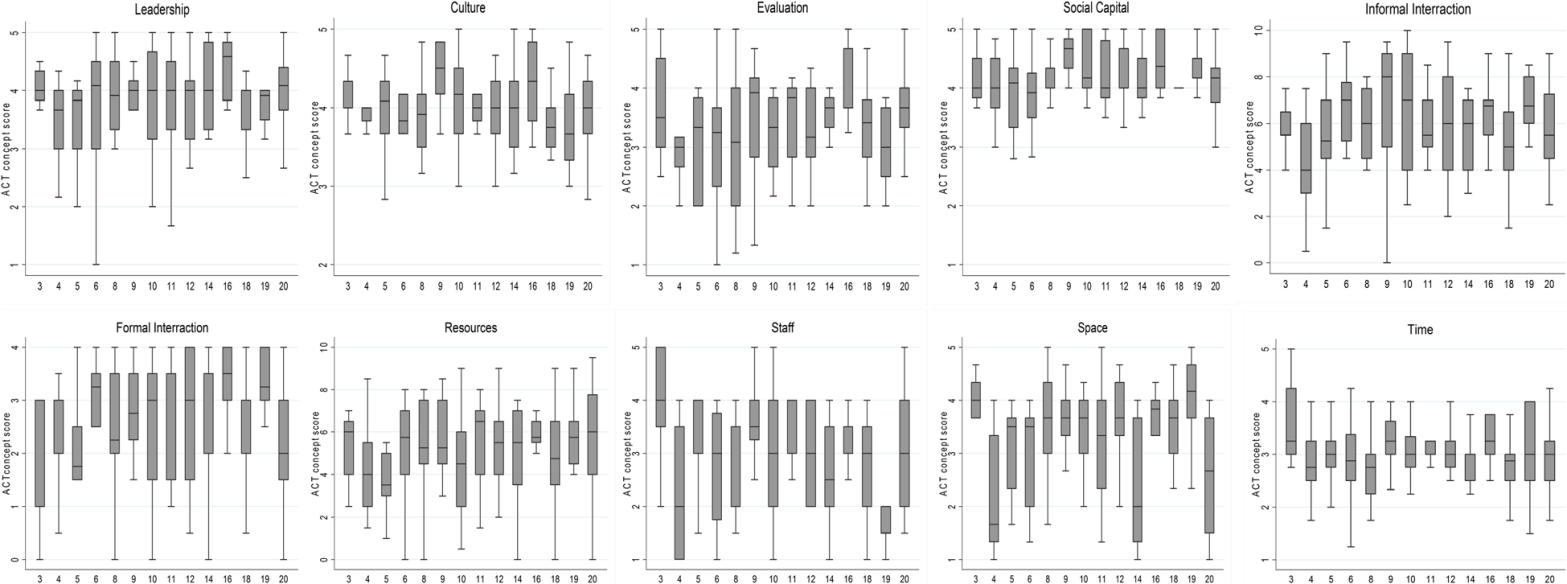
Unit level variations in Alberta Context Tool dimensions. *Excludes units in which < 8 staff provided responses (*N* = 5)

Variations existed between and within units for mean ACT concept scores (Fig. 1). Between-unit differences were statistically significant for the concepts of evaluation, space, and staff. The level of aggregation at the unit level was acceptable for all concepts except structural/electronic resources and time. Variations in unit-level aggregation were also observed between responses for nurses and allied health/medical staff for the different items (Table 3), but overall mean concept scores were similar between groups (Table 3). Also, there were few differences in the ten ACT concepts and project-specific survey responses between metropolitan and regional sites.

**Table 3:** Mean Alberta Context Tool concept scores and intraclass correlations, overall and by profession

Concept	ICC, all^#^	ICC AH/Med	ICC, nursing	ACT score, all^#^ Mean (SD), *N* = 215	ACT score AH/Med Mean (SD), *N* = 98	ACT score, nursing Mean (SD), *N* = 105	*p* value
1. Leadership	0.17	0.21	0.13	3.82 (0.80)	3.74 (0.87)	3.89 (0.73)	0.25
2. Culture	0.13	0.14	0.01	3.99 (0.51)	4.03 (0.55)	3.95 (0.49)	0.20
3. Evaluation	0.16	0.25	0.04	3.36 (0.82)	3.33 (0.84)	3.37 (0 .81)	0.87
4. Social capital	0.14	0.32	0.00	4.14 (0.59)	4.17 (0.60)	4.10 (0.59)	0.49
5. Informal interactions	0.11	0.10	0.08	5.80 (2.13)	5.80 (1.99)	5.75 (2.30)	0.87
6. Formal interactions	0.13	0.10	0.18	2.54 (1.12)	2.69 (1.08)*	2.37 (1.14)*	0.05
7. Resources	0.07	0.08	0.19	5.17 (2.11)	5.02 (1.91)	5.17 (2.24)	0.29
Organizational slack							
8. Staff	0.17	0.03	0.31	3.00 (1.01)	3.06 (0.98)	2.99 (1.05)	0.74
9. Space	0.24	0.21	0.25	3.22 (1.06)	3.34 (1.00)	3.13 (1.08)	0.27
10. Time	0.08	0.01	0.13	3.02 (0.68)	3.08 (0.62)	2.99 (0.71)	0.27

*ICC* intraclass correlation, *SD* standard deviation, *AH* allied health, *Med* medical profession

^#^Includes responses from other professions (*N* = 6) and those with missing profession (*N* = 7)

*Statistically significant difference (*p* < 0.05)

**Fig. 2:**
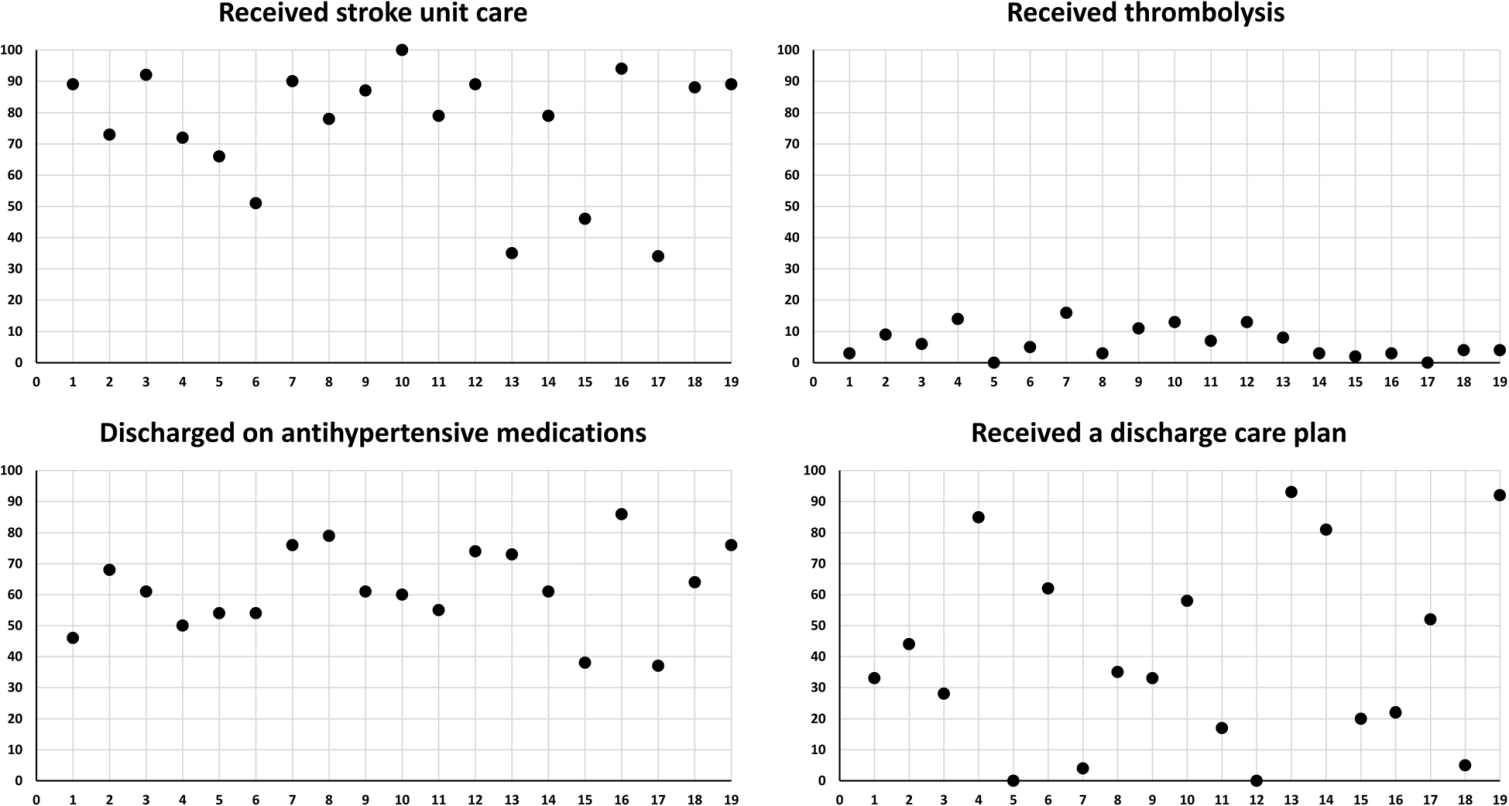
Variations in adherence to care processes by the hospital

The proportion of patients who received recommended care also varied widely between hospitals: 34 to 100% for stroke unit care, 0 to 16% for thrombolysis, 37 to 86% for prescription of antihypertensive medication at discharge, and 0 to 93% for discharge care planning (Fig. 2). Table 4 shows that seven of the ACT concepts were significantly associated with a greater proportion of patients receiving stroke unit care. Of these, social capital (μ 9.00, 95% confidence interval [CI] 4.86 to 13.14) and culture (μ 7.33, 95% CI 2.05 to 12.62) had the strongest association; associations were predominantly driven by responses from the allied health/medical staff. Thrombolysis treatment for AIS patients and prescription of antihypertensive medications at discharge were not significantly associated with any of the ACT concepts. Receipt of a discharge care plan for those discharged to home was significantly but negatively associated with space.

**Table 4:** Association between Alberta Context Tool concept scores and the proportion of eligible patients receiving recommended stroke care

	Received stroke unit care			Received thrombolysis*		
	All	Nursing	AH/Med	All	Nursing	AH/Med
	Co-efficient (95% CI)	Co-efficient (95% CI)	Co-efficient (95% CI)	Co-efficient (95% CI)	Co-efficient (95% CI)	Co-efficient (95% CI)
1. Leadership	3.20 (− 0.42, 6.82)	0.71 (− 2.12, 3.55)	0.67 (− 4.40, 5.73)	0.00 (− 0.72, 0.72)	0.00 (− 1.18, 1.18)	− 0.46 (− 2.35, 1.43)
2. Culture	*7*.*33* (*2*.*05*, *12*.*62*)	1.0 (− 3.64, 5.64)	*11*.*00* (*5*.*35*, *16*.*65*)	1.50 (− 0.44, 3.44)	1.50 (− 0.06, 3.06)	0.00 (− 3.02, 3.02)
3. Evaluation	*4*.*36* (*0*.*99*, *7*.*74*)	0.55 (− 2.12, 3.21)	*9*.*33* (*4*.*06*, *14*.*6*)	− 0.35 (− 1.13, 0.42)	0.00 (− 0.84, 0.84)	− 0.75 (− 2.64, 1.14)
4. Social capital	*9*.*00* (*4*.*86*, *13*.*14*)	1.20 (− 3.46, 5.89)	*10*.*77* (*5*.*95*, *1*.*59*)	1.00 (− 0.54, 2.54)	0.67 (− 0.36, 1.69)	0.56 (− 2.11, 3.22)
5. Informal interactions	*1*.*80* (*0*.*21*, *3*.*39*)	0.14 (− 0.84, 1.13)	*2*.*67* (*0*.*56*, *4*.*77*)	0.13 (− 0.24, 0.51)	0.25 (− 0.14, 0.64)	− 0.44 (−1.58, 0.69)
6. Formal interactions	0.00 (− 1.75, 1.75)	− 0.5 (− 2.79, 1.79)	*4*.*50* (*0*.*09*, *8*.*91*)	0.00 (− 0.53, 0.53)	0.00 (− 0.62, 0.62)	− 1.33 (− 2.83, 0.17)
7. Resources	1.11 (− 0.12, 2.34)	0.33 (− 1.10, 1.77)	2.25 (− 0.02, 4.52)	0.00 (− 0.31, 0.31)	0.00 (− 0.40, 0.40)	− 0.67 (− 1.74, 0.40)
Organizational slack						
8. Staff	*3*.*67* (*1*.*09*, *6*.*24*)	0.50 (− 1.63, 2.63)	2.57 (− 1.40, 6.54)	0.57 (− 0.01, 1.15)	0.40 (− 0.26, 1.06)	0.67 (− 0.97, 2.31)
9. Space	*3*.*00* (*0*.*44*, *5*.*56*)	0.00 (− 2.12, 2.12)	*4*.*13* (*0*.*24*, *8*.*01*)	0.50 (− 0.08, 1.08)	0.38 (− 0.24, 0.99)	0.55 (− 0.91, 2.00)
10.Time	*4*.*57* (*1*.*17*, *7*.*97*)	3.33 (− 0.69, 7.35)	5.14 (− 2.21, 12.49)	0.80 (− 0.06, 1.70)	0.50 (− 0.29, 1.29)	1.00 (− 1.63, 3.63)
	Discharged on antihypertensives			Discharge care plan provided^#^		
	All	Nursing	AH/Med	All	Nursing	AH/Med
	Co-efficient (95% CI)	Co-efficient (95% CI)	Co-efficient (95% CI)	Co-efficient (95% CI)	Co-efficient (95% CI)	Co-efficient (95% CI)
1. Leadership	2.25 (− 0.97, 5.47)	0.00 (− 7.64, 7.64)	2.40 (− 2.02, 6.82)	0.00 (− 6,92, 6.92)	7.13 (− 5.92, 20.17)	− 0.75 (−10.78, 9.28)
2. Culture	0.00 (− 5.10, 5.10)	− 1.8 (− 10.97, 7.37)	2.00 (− 4.88, 8.88)	− 12.67 (− 25.56, 0.23**)**	0.00 (− 20.91, 20.91)	− 14.73 (− 31.32, 1.87)
3. Evaluation	0.86 (− 2.48, 4.20)	0.00 (− 6.73, 6.73)	3.50 (− 0.97, 7.97)	− 4.5 (− 10.99, 1.99)	6.67 (− 5.89, 19.22)	− 7.80 (− 18.65, 3.05)
4. Social capital	0.00 (− 4.52, 4.52)	0.00 (− 12.86, 12.86)	2.53 (− 2.47, 7.53)	− 9.43 (− 22.24, 3.38)	0.00 (−19.49, 19.49)	− 10.38 (− 23.29, 2.52)
5. Informal interactions	0.00 (− 1.20, 1.20)	0.00 (− 2.13, 2.13)	0.00 (− 1.74, 1.74)	− 2.38 (− 5.79, 1.04)	0.00 (− 4.43, 4.43)	0.00 (− 6.84, 6.84)
6. Formal interactions	0.00 (− 2.19, 2.19)	− 2.67 (− 5.44, 0.11)	0.50 (− 3.06, 4.06)	− 6.25 (− 14.37, 1.87)	0.00 (− 8.93, 8.93)	− *9*.*00* (− *15*.*91*, − *2*.*09*)
7. Resources	0.33 (− 0.97, 1.63)	0.40 (− 1.66, 2.46)	0.50 (− 1.30, 2.30)	− 2.11 (− 5.06, 0.84)	0.00 (− 5.05, 5.05)	0.00 (− 6.21, 6.21)
Organizational slack						
8. Staff	0.00 (− 2.49, 2.49)	− 1.00 (− 4.52, 2.52)	0.00 (− 3.94, 3.94)	− 7.50 (− 15.06, 0.06)	0.00 (− 11.23, 11.23)	− 5.0 (− 17.47, 7.47)
9. Space	0.00 (− 2.91, 2.91)	− 1.29 (− 4.55, 1.98)	2.50 (− 1.32, 6.32)	− *16*.*00* (− *19*.*99*, − *12*.*01*)	− *17*.*40* (− *23*.*01*, − *11*.*79*)	− *11*.*0* (− *21*.*39*, − *0*.*61*)
10.Time	0.00 (− 3.71, 3.71)	0.00 (− 6.82, 6.82)	0.00 (− 6.19, 6.19)	− 9.50 (− 18.47, − 0.53)	0.00 (− 16.46, 16.46)	− 7.71 (− 20.34, 4.91)

Italicized values are statistically significant at *p* < 0.05

*ICC* interclass correlation, *AH* allied health, *Med* medical profession, *CI* confidence interval

*Includes only those with ischemic stroke

^#^Includes only those discharged directly to the community from acute care

**Fig. 3:**
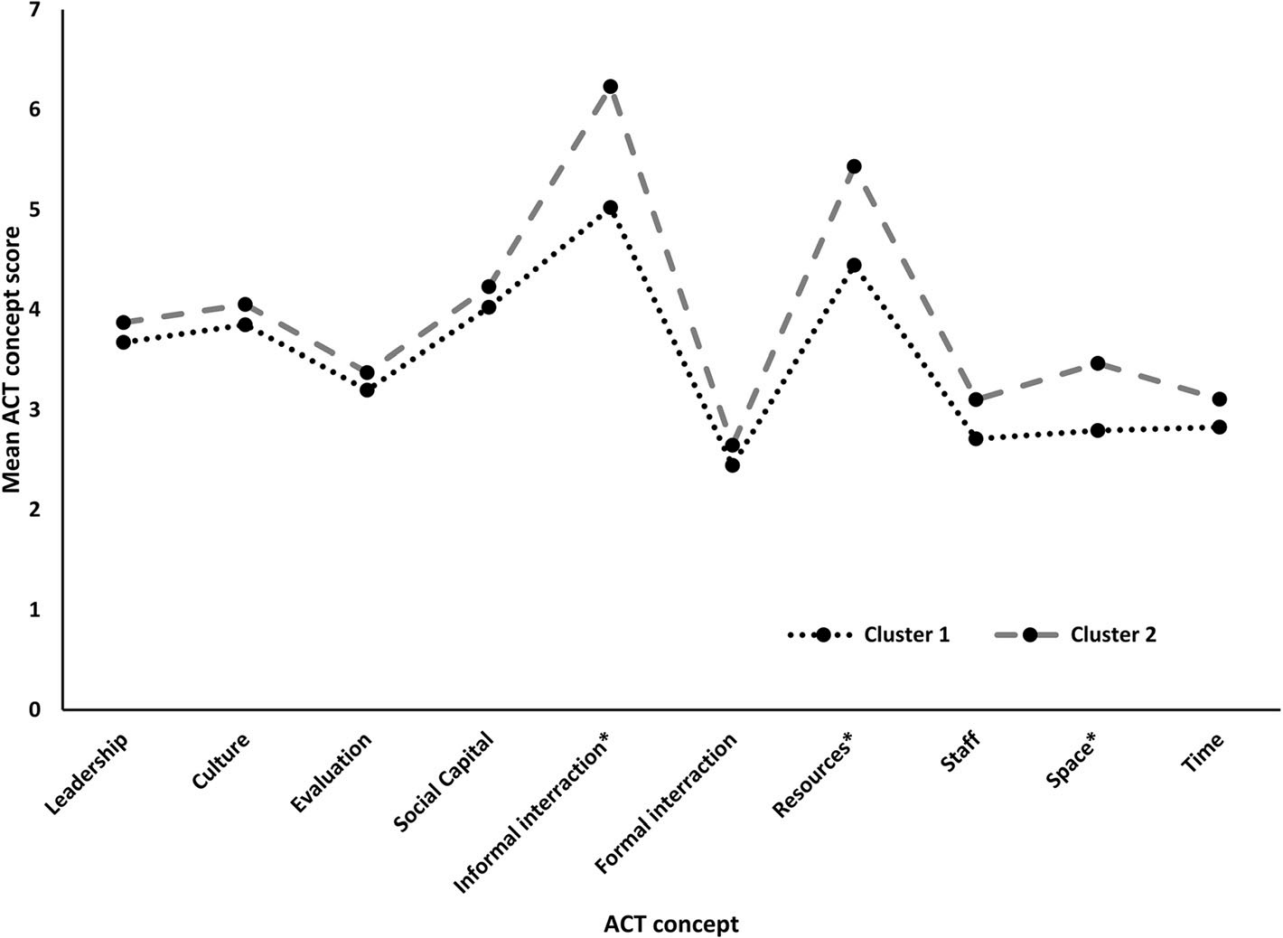
Alberta Context Tool concept scores by cluster groupings. Asterisk indicates a statistically significant difference between clusters for that concept. ACT, Alberta Context Tool. Cluster 1 = lower context, cluster 2 = higher context

Cluster analysis identified two distinct clusters based on responses to all ten ACT concepts (Fig. 3). Mean scores from units in cluster 1 (*n* = 4) were lower than those for units in cluster 2 (*n* = 10) for all concepts. These differences were statistically significant for the concepts of informal interactions, structural/electronic resources, and space (Fig. 3). Overall, units in cluster 2 had a significantly greater proportion of patients that were treated on a stroke unit (cluster 1 69.0% vs. cluster 2 88.5%; *p* = 0.03), and significantly, more patients were discharged on antihypertensive medications compared to those in cluster 1 (cluster 1 52.0% vs. cluster 2 62.5%; *p* = 0.03) (Table 5).

**Table 5:** Proportion of patients who received stroke quality of care processes by level of overall context

Overall hospital level context*	Received stroke unit care	Received thrombolysis^†^	Discharged on antihypertensives	Discharge care plan provided^#^
	Median (Q1, Q3)	Median (Q1, Q3)	Median (Q1, Q3)	Median (Q1, Q3)
Cluster 1 (lower context), *N* = 4	69.0 (50.0, 75.5)	1.5 (0, 8.5)	52.0 (43.5, 57.5)	66.5 (26.0, 83.0)
Cluster 2 (higher context), *N* = 10	88.5 (64.5, 92.0)	5.5 (4, 11)	62.5 (60.0, 76.0)	30.5 (17.0, 58.0)
*p* value	0.03	0.20	0.03	0.44

*Q1* quartile 1, *Q3* quartile 3

*Overall context grouping was determined using exploratory cluster analysis to categorize units into two clusters based on responses to the Alberta Context Tool and excludes sites with < 8 responses (*N* = 5)

^†^Includes only patients with ischemic stroke

^#^Includes only patients discharged directly to the community from acute care

Analysis of the open-ended responses and survey part 1 responses (Table 2) showed that while staff reported that having a multidisciplinary team and dedicated staff were a strength, those working within higher (cluster 2) compared to lower (cluster 1) context units were more likely to report that medical and nursing staff engagement and leadership in the form of a stroke care coordinator facilitated stroke care. Staff from units working within a higher context were also more likely to indicate that the use of processes, protocols, or pathways was “a strength” compared to staff working in the lower context units. Those in higher context units were significantly more likely to report that the introduction of protocols had been effective and that staff were familiar with these protocols (Table 2).

Three major themes were identified from the qualitative results: team structures and communication, leadership, and processes. Common barriers reported by staff across most hospitals were a lack of staff and/or trained staff and a lack of time. Lack of a medical lead or medical staff engagement, poor communication across disciplines, and lack of staff with stroke-specific training were a common theme among clinicians from units working within a lower context. Lack of support or coordination with the emergency department was mentioned as a barrier by staff across most sites, especially in regard to the delivery of thrombolysis. A common enabler was having a committed multidisciplinary team. However, those from high context sites were more likely to report using performance monitoring and strong leadership as enablers (Additional file 4: Table S3).

## Discussion

Our study provides new evidence of the relationship between perceived organizational contextual factors and delivery of acute stroke care. Aspects of organizational context measured using the ACT were significantly associated with a greater proportion of patients receiving stroke unit care but had less impact on the delivery of the other quality parameters. On average, the proportions of patients that received stroke unit care and prescribed antihypertensive medication at discharge were some 10 to 20% greater in sites clustered according to having a higher context compared to those in the lower context group.

Stroke unit care is recommended for all patients with acute stroke, as it involves the delivery of a range of recommended interventions through coordinated multidisciplinary team care [27]. However, effective access depends on integration and cooperation across multiple departments, bed management services, and staff. Social capital emerged as the dominant feature both within the ACT results pertaining to stroke unit care and the qualitative results. As with context, there is no agreed definition of social capital. However, a common feature is the focus on social relations that have productive benefits. Establishing unit-based multidisciplinary teams, optimizing staff stability, and promoting effective team-based leadership are modifiable strategies that may positively impact on social capital to improve knowledge translation. Strong social capital may also promote knowledge translation through team learning and the sharing of knowledge and expertise between colleagues of different disciplines.

A lower level of association was evident between organizational context and the delivery of some other aspects of stroke care. The delivery of thrombolysis is complex and highly staff-dependent with a short time window from the onset of symptoms of AIS [7]. The treatment, therefore, requires good coordination beyond the organizational unit, including between ambulance services and the emergency department [28]. This was reinforced in the responses to several of the open-ended questions in which many clinicians felt that barriers to delivery of this type of care were often external to their unit.

Individual ACT concepts measured at the clinician level did not have a significant positive association with either of the discharge care processes. However, greater overall context at a unit level was significantly associated with the process variable being discharged on antihypertensive medications. Results pertaining to receipt of a discharge care plan were inconsistent and may reflect the greater level of variability in adherence to this indicator compared to the other care processes [5].

Our study is one of the few that have used the ACT to examine the multidisciplinary organizational context. Most of the prior literature on the delivery of acute hospital care has focused specifically on nursing staff [20, 29–31]. We had expected that context scores provided by nursing staff located in a single unit would have differed to those of allied health or medical staff who often work across multiple units. There was little difference between these groups. This may be a feature of stroke care and particularly care provided within stroke units in which staff are likely to work collaboratively within a single unit across disciplines. The importance of multidisciplinary care was confirmed in responses to the survey. Contrary to other studies, the organizational context of stroke unit care is likely to be dependent on the contributions within and between disciplines [27]. Consistent with the recent literature, leadership within the unit was also considered by respondents to be important for successful delivery of evidence-based care [32].

Similar to other studies, the mean organizational context reported by participants in our study was generally high [29–31]. While the ACT had acceptable reliability and validity similar to other studies performed in Australia [31], the unit level variability was generally less in our study [30, 31, 33]. The strong outer contextual support for knowledge translation provided in Australia through Clinical Guidelines [4], biennial-independent audits [3], and systems of continuous data collection [34] may have reduced variation in delivery of care. In particular, Queensland, the Australian state in which the Stroke123 study was undertaken, has a long history of quality improvement activities facilitated through the Queensland Statewide Stroke Clinical Network and the Stroke Foundation’s StrokeLink program [16]. Through biannual meetings, the Queensland Statewide Stroke Clinical Network examines hospital performance data and agrees to state-level quality improvement priorities and strategies. In addition, biennial externally facilitated workshops (StrokeLink) are offered at hospitals (usually one per hospital) to assist with dissemination of evidence and action planning to improve the application of knowledge by addressing areas of sub-optimal performance based on data from AuSCR and national audits. Staff from hospitals participating in our study were actively involved in these initiatives and reported that the Queensland Statewide Stroke Clinical Network had positively influenced their practice.

We recognize some limitations to our study. Variations in the number of staff per site that provided responses to the survey may have influenced the extent to which the responses represented the perception of context for the unit as a whole. We tried to minimize this bias by excluding sites that had less than eight responses when aggregating results at a unit level, and the good internal reliability both overall and when stratified by profession provides some reassurance that this was achieved. As there were only a small number of hospitals within the low context cluster, and none of these were regional hospitals, we were unable to make comparisons between these metropolitan and regional locations based on clusters. Another limitation is the likelihood of missed or chance associations as the sample size was determined for the main Stroke123 study [23]. This issue is especially relevant to the delivery of thrombolysis as there were small numbers of patients with AIS who received this process variable. Finally, our study may be limited in its ability to be generalized to other hospitals in Australia or other countries.

## Conclusion

Our study offers insights into the potential contextual mechanisms that may be leveraged for improving implementation of evidence into practice from the perspective of acute stroke care. Organizational context, as measured by the ACT, was important in delivering acute stroke care processes, although the strength of association varied across the different components with aspects of care, particularly those related to hospital discharge plans. These data add insight into contextual factors related to interpreting the findings of the Stroke123 project.

## Additional files

Additional file 1: Project specific survey. (PDF 228 kb)

Additional file 2: **Table S1.** Demographic characteristics of participating clinicians. (DOCX 21 kb)

Additional file 3: **Table S2.** Alberta Context Tool reliability results overall and by profession. (DOCX 16 kb)

Additional file 4: **Table S3.** Qualitative results: perceived barriers and facilitators to delivering acute stroke care. (DOCX 14 kb)

Additional file 5: Supplemental acknowledgements. List of project contributors not included in the Acknowledgements section of the main manuscript (PDF 85 kb)

## Abbreviations

ACT: Alberta Context Tool
AIS: Acute ischemic stroke
AuSCR: Australian Stroke Clinical Registry
CFA: Confirmatory factor analysis
ICC: Interclass correlation
PARIHS: Promoting Action on Research Implementation in Health Services
rtPA: Recombinant tissue plasminogen activator
TIA: Transient ischemic attack
