# Correction to: Hospital organizational context and delivery of evidence-based stroke care: a cross-sectional study

**DOI:** 10.1186/s13012-019-0862-x

**Published:** 2019-02-08

**Authors:** Nadine E. Andrew, Sandy Middleton, Rohan Grimley, Craig S. Anderson, Geoffrey A. Donnan, Natasha A. Lannin, Enna Stroil-Salama, Brenda Grabsch, Monique F. Kilkenny, Janet E. Squires, Dominique A. Cadilhac, On behalf of the Stroke123 Investigators

**Affiliations:** 1 Stroke and Ageing Research, Department of Medicine, School of Clinical Sciences at Monash Health, Monash University, Level 3, Hudson Institute Building, 27-31 Wright Street, Clayton, VIC 3168 Australia; 2 Department of Medicine, Peninsula Clinical School, Central Clinical School, Monash University, Melbourne, Australia; 3 Nursing Research Institute, St Vincent’s Health Australia (Sydney) and Australian Catholic University, Sydney, Australia; 4 Sunshine Coast Clinical School, The University of Queensland, Birtinya and Statewide Stroke Clinical Network, Queensland Health, Brisbane, Australia; 5 The George Institute for Global Health, Faculty of Medicine, University of New South Wales, Sydney, Australia; 6 Florey Institute of Neuroscience and Mental Health, University of Melbourne, Heidelberg, Australia; 7 Faculty of Health Sciences, La Trobe University, Bundoora, Australia; 8 Occupational Therapy Department, Alfred Health, Melbourne, Australia; 9 Australian Bronchiectasis Registry, Lung Foundation Australia, Brisbane, Australia; 10 School of Nursing, Faculty of Health Sciences, University of Ottawa, Ottawa, Ontario Canada

**Correction to: Implement Sci (2019) 14:6**

**https://doi.org/10.1186/s13012-018-0849-z**

Following publication of the original article [1], the authors reported an error in one of the authors’ names. In this Correction the incorrect and correct author name are shown. The original article has been corrected.

Originally the author name was published as:

- Enna Striol-Salama

The correct author name is:

- Enna Stroil-Salama
