# Supplementary material for: Hospital organizational context and delivery of evidence-based stroke care: a cross-sectional study
Source: Implement Sci. 2019 Jan 18;14:6. doi: 10.1186/s13012-018-0849-z (PMC6339367; doi:10.1186/s13012-018-0849-z)
Supplement: Supplementary file 1 — Project specific survey. (PDF 228 kb) [file 13012_2018_849_MOESM1_ESM.pdf]

STAFF SURVEY

To be completed in addition to the Alberta Context Tool

If you work at multiple hospitals please answer this questionnaire with regards to where you are working today.

Survey ID:________________ Hospital ID:_______________

Date survey completed: DD/MM/20YY

Profession:
- ☐ DOCTOR
- ☐ PHYSIOTHERAPIST
- ☐ OCCUPATIONAL THERAPIST
- ☐ NURSE
- ☐ SPEECH PATHOLOGIST
- ☐ DIETITIAN
- ☐ ADMINISTRATOR/EXECUTIVE
- ☐ OTHER____________________

Please mark your *completed* education program(s). **(Select all that apply)**

- ☐ Diploma/Certificate
- ☐ Bachelors Degree
- ☐ Medical Degree
- ☐ Masters Degree (in related area)
- ☐ Doctorate

Current role at this hospital:____

Number of months in current role:___________________________________

Age______________________

Gender: ☐ Male ☐ Female

Employment status: ☐ Full Time ☐ Part Time

Department / Unit:_______________________________

MANAGEMENT INVOLVEMENT

	Strongly Disagree	Disagree	Neither Agree Nor Disagree	Agree	Strongly Agree
1. There is adequate support from executive staff to achieve plans for improving stroke care within this hospital	☐	☐	☐	☐	☐
2. Robust two way communication processes are in place between staff and management across all levels of my organisation	☐	☐	☐	☐	☐

	Strongly Disagree	Disagree	Neither Agree Nor Disagree	Agree	Strongly Agree
3. Regular, understandable updates are provided by management across all levels of my organisation	☐	☐	☐	☐	☐

TEAMWORK / STAFF

	Strongly Disagree	Disagree	Neither Agree Nor Disagree	Agree	Strongly Agree
4. Multidisciplinary communication across the hospital facilitates effective care for stroke at this hospital	☐	☐	☐	☐	☐
5. My unit has a stable workforce the majority of the time (ie. limited use of agency or short term casual staff)	☐	☐	☐	☐	☐
6. Staff are familiar with stroke protocols	☐	☐	☐	☐	☐
7. The level of **medical** staff engagement facilitates an ability to improve stroke care at this hospital	☐	☐	☐	☐	☐
8. The level of **allied health** staff engagement facilitates an ability to improve stroke care at this hospital	☐	☐	☐	☐	☐
9. The level of **nursing** staff engagement facilitates an ability to improve stroke care at this hospital	☐	☐	☐	☐	☐

ORGANISATIONAL CHANGE

	Strongly Disagree	Disagree	Neither Agree Nor Disagree	Agree	Strongly Agree
10. I have sufficient opportunities to question managers about change at work	☐	☐	☐	☐	☐
11. Staff are always consulted about change at work	☐	☐	☐	☐	☐

12. When changes are made at work, I am clear how they will work out in practice	Strongly Disagree ☐	Disagree ☐	Neither Agree Nor Disagree ☐	Agree ☐	Strongly Agree ☐

13. The introduction of protocols or care pathways for stroke has been effective for improving care for patients with stroke	Strongly Disagree ☐	Disagree ☐	Neither Agree Nor Disagree ☐	Agree ☐	Strongly Agree ☐	Not applicable ☐

14. Access to patient and family education about stroke provided by hospital staff is adequate to meet their needs	Strongly Disagree ☐	Disagree ☐	Neither Agree Nor Disagree ☐	Agree ☐	Strongly Agree ☐

OTHER

15. The Queensland Health Stroke Services Clinical Network has had a positive impact on stroke care within my hospital	Strongly Disagree ☐	Disagree ☐	Neither Agree Nor Disagree ☐	Agree ☐	Strongly Agree ☐
16. The Queensland Health Stroke Services Clinical Network has had a positive impact on stroke care within my area health network					
17. Professional development opportunities (including attending education or in-service sessions) for clinical staff has improved knowledge about clinical care for stroke at this hospital	Strongly Disagree ☐	Disagree ☐	Neither Agree Nor Disagree ☐	Agree ☐	Strongly Agree ☐

18. What type of performance information do you currently use for quality improvement planning and activities? **(tick all that apply)**

☐ None
☐ Intermittent internal audits
☐ Regular internal audits
☐ Access to continuous feedback via internal stroke register or AuSCR
☐ Queensland Stroke Services Clinical Network data
☐ National Stroke Foundation audit
☐ Other (Please Specify)___________________________________

19. Have you been directly involved in a quality improvement initiative or program for improving stroke care at this hospital?

☐ Yes
☐ No

If yes, was this undertaken: internally by

☐ local staff
☐ an external facilitator
☐ both
