# Supplementary material for: Hospital organizational context and delivery of evidence-based stroke care: a cross-sectional study
Source: Implement Sci. 2019 Jan 18;14:6. doi: 10.1186/s13012-018-0849-z (PMC6339367; doi:10.1186/s13012-018-0849-z)
Supplement: Supplementary file 2 — Table S1. Demographic characteristics of participating clinicians. (DOCX 21 kb) [file 13012_2018_849_MOESM2_ESM.docx]

**Supplemental Table 1. Demographic characteristics of participating clinicians**

|  |  | **Hospital ID number**  **n (%)** | | | | | | | | | | | | | | | | | | | |
| --- | --- | --- | --- | --- | --- | --- | --- | --- | --- | --- | --- | --- | --- | --- | --- | --- | --- | --- | --- | --- | --- |
|  | **Total**  **(N)** | **1**  **(4)** | **2**  **(7)** | **3**  **(11)** | **4**  **(11)** | **5**  **(11)** | **6**  **(13)** | **7**  **(6)** | **8**  **(10)** | **9**  **(12)** | **10**  **(15)** | **11**  **(11)** | **12**  **(19)** | **13**  **(7)** | **14**  **(11)** | **15**  **(3)** | **16**  **(10)** | **17**  **(18)** | **18**  **(10)** | **19**  **(23)** | **p value** |
| **Age group** |  |  |  |  |  |  |  |  |  |  |  |  |  |  |  |  |  |  |  |  |  |
| *<30* | 51  (25) | 0  (0) | 3  (43) | 2  (20) | 2  (20) | 3  (30) | 3  (25) | 2  (33) | 1  (11) | 1  (8) | 6  (43) | 2  (25) | 4  (21) | 2  (29) | 2  (18) | 0  (0) | 3  (30) | 4  (22) | 4  (44) | 7  (25) |  |
| *31-39* | 50  (24) | 1  (25) | 2  (29) | 3  (30) | 2  (20) | 2  (20) | 5  (42) | 1  (17) | 4  (44) | 2  (17) | 1  (7) | 1  (13) | 3  (16) | 2  (29) | 3  (27) | 0  (0) | 4  (40) | 2  (11) | 3  (33) | 9  (32) | 0.5 |
| *40-49* | 56  (27) | 1  (25) | 0  (0) | 1  (10) | 2  (20) | 4  (40) | 3  (25) | 0  (0) | 2  (22) | 5  (42) | 5  (36) | 1  (13) | 10  (53) | 1  (14) | 2  (18) | 1  (33) | 2  (20) | 9  (50) | 1  (11) | 6  (21) |  |
| *50+* | 50  (24) | 2  (50) | 2  (29) | 4  (40) | 4  (40) | 1  (10) | 1  (8) | 3  (50) | 2  (22) | 4  (33) | 2  (14) | 4  (50) | 2  (11) | 2  (29) | 4  (36) | 2  (67) | 1  (10) | 3  (17) | 1  (11) | 6  (21) |  |
|  |  |  |  |  |  |  |  |  |  |  |  |  |  |  |  |  |  |  |  |  |  |
| **Female** | 160  (78) | 2  (50) | 3  (50) | 7  (70) | 8  (80) | 10  (100) | 10  (83) | 4  (67) | 8  (89) | 11  (92) | 12  (86) | 5  (63) | 13  (68) | 4  (57) | 9  (82) | 3  (100) | 7  (70) | 13  (72) | 8  (89) | 23  (82) | 0.5 |
| **Profession** |  |  |  |  |  |  |  |  |  |  |  |  |  |  |  |  |  |  |  |  |  |
| *Doctor* | 21  (10) | 1  (25) | 1  (14) | 0  (0) | 2  (20) | 1  (10) | 1  (8) | 1  (17) | 1  (11) | 1  (8) | 1  (7) | 1  (11) | 2  (11) | 3  (43) | 1  (9) | 0  (0) | 2  (20) | 0  (0) | 1  (11) | 1  (4) |  |
| *Allied Health* | 77  (37) | 2  (50) | 2  (29) | 4  (40) | 4  (40) | 7  (70) | 4  (33) | 1  (17) | 4  (44) | 5  (42) | 7  (50) | 4  (44) | 9  (47) | 3  (43) | 4  (36) | 0  (0) | 4  (40) | 5  (28) | 5  (56) | 3  (11) | 0.06 |
| *Nurse* | 105  (50) | 1  (25) | 4  (57) | 6  (60) | 3  (30) | 2  (20) | 7  (58) | 4  (67) | 4  (44) | 5  (42) | 6  (43) | 4  (44) | 8  (42) | 0  (0) | 4  (36) | 3  (100) | 4  (40) | 13  (72) | 3  (33) | 24  (86) |  |
| *Other* | 5  (2) | 0  (0) | 0  (0) | 0  (0) | 1 (10) | 0  (0) | 0  (0) | 0  (0) | 0  (0) | 1  (8) | 0  (0) | 0  (0) | 0  (0) | 1  (14) | 2  (18) | 0  (0) | 0  (0) | 0  (0) | 0  (0) | 0  (0) |  |
| **Education** |  |  |  |  |  |  |  |  |  |  |  |  |  |  |  |  |  |  |  | | |
| *Diploma* | 20  (10) | 1  (25) | 1  (14) | 1  (10) | 0  (0) | 0  (0) | 0  (0) | 0  (0) | 0  (0) | 2  (17) | 1  (7) | 1  (11) | 0  (0) | 1  (14) | 0  (0) | 2  (67) | 0  (0) | 4  (22) | 0  (0) | 6  (21) |  |
| *Bachelor* | 144  (69) | 2  (50) | 4  (57) | 6  (60) | 7  (70) | 8  (80) | 9  (75) | 5  (83) | 5  (56) | 9  (75) | 9  (64) | 7  (78) | 17  (89) | 3  (43) | 9  (82) | 1  (33) | 5  (50) | 13  (72) | 4  (44) | 21  (75) | 0.02 |
| *Medical* | 19  (9) | 1  (25) | 1  (14) | 0  (0) | 1  (10) | 1  (10) | 1  (8) | 1  (17) | 1  (11) | 1  (8) | 0  (0) | 1  (11) | 2  (11) | 2  (29) | 1  (9) | 0  (0) | 3  (30) | 0  (0) | 1  (11) | 1  (4) |  |
| *Masters or PhD* | 25  (12) | 0  (0) | 1  (14) | 3  (30) | 2  (20) | 1  (10) | 2  (17) | 0  (0) | 3  (33) | 0  (0) | 4  (29) | 0  (0) | 0  (0) | 1  (14) | 1  (9) | 0  (0) | 2  (20) | 1  (6) | 4  (44) | 0  (0) |  |
| **Time in role** |  |  |  |  |  |  |  |  |  |  |  |  |  |  |  |  |  |  |  |  |  |
| *< 1 year* | 40  (19) | 0  (0) | 1  (14) | 0  (0) | 1  (9) | 4  (40) | 2  (17) | 2  (33) | 3  (33) | 1  (8) | 2  (14) | 1  (9) | 6  (32) | 2  (29) | 1  (9) | 0  (0) | 6  (60) | 3  (17) | 3  (30) | 2  (7) |  |
| *1-2 years* | 55  (26) | 1  (25) | 1  (14) | 2  (18) | 4  (36) | 4  (40) | 3  (25) | 1  (17) | 4  (44) | 2  (17) | 2  (14) | 1  (9) | 6  (32) | 3  (43) | 6  (55) | 0  (0) | 0  (0) | 4  (22) | 4  (40) | 7  (25) | 0.02 |
| *3-5 years* | 45  (21) | 1  (25) | 4  (57) | 3  (27) | 3  (27) | 1  (10) | 2  (17) | 1  (17) | 0  (0) | 1  (8) | 8  (57) | 2  (18) | 4  (21) | 1  (14) | 1  (9) | 2  (67) | 1  (10) | 4  (22) | 0  (0) | 6  (21) |  |
| *>5 years* | 73  (34) | 2  (50) | 1  (14) | 6  (55) | 3  (27) | 1  (10) | 5  (42) | 2  (33) | 2  (22) | 8  (67) | 2  (14) | 7  (64) | 3  (16) | 1  (14) | 3  (27) | 1  (33) | 3  (30) | 7  (39) | 3  (30) | 13  (46) |  |
