# Supplementary material for: Hospital organizational context and delivery of evidence-based stroke care: a cross-sectional study
Source: Implement Sci. 2019 Jan 18;14:6. doi: 10.1186/s13012-018-0849-z (PMC6339367; doi:10.1186/s13012-018-0849-z)
Supplement: Supplementary file 3 — Table S2. Alberta Context Tool reliability results overall and by profession. (DOCX 16 kb) [file 13012_2018_849_MOESM3_ESM.docx]

**Supplemental Table 2. Alberta Context Tool reliability overall and by profession**

| **ACT Concept** | **α^#^** | **Average Item-rest correlations*** | **Average Inter-item correlation^** | **α^#^** | **Average Item-rest correlation*** | **Average Inter-item correlation^** | **α^#^** | **Average Item-rest correlation*** | **Average Inter-item correlation^** |
| --- | --- | --- | --- | --- | --- | --- | --- | --- | --- |
|  | **All staff**  **N=215^§^** | | | **Nursing staff**  **N=105** | | | **Allied health or Medical staff**  **N=97** | | |
| Leadership | 0.81 | 0.47 | 0.33 | 0.81 | 0.36 | 0.32 | 0.82 | 0.57 | 0.34 |
| Culture | 0.80 | 0.64 | 0.30 | 0.78 | 0.67 | 0.28 | 0.82 | 0.65 | 0.33 |
| Evaluation | 0.81 | 0.53 | 0.32 | 0.80 | 0.49 | 0.30 | 0.82 | 0.55 | 0.34 |
| Social capital | 0.80 | 0.63 | 0.30 | 0.78 | 0.61 | 0.28 | 0.81 | 0.70 | 0.32 |
| Informal interactions | 0.81 | 0.53 | 0.32 | 0.80 | 0.50 | 0.30 | 0.82 | 0.57 | 0.34 |
| Formal interactions | 0.82 | 0.45 | 0.33 | 0.80 | 0.43 | 0.31 | 0.83 | 0.48 | 0.35 |
| Resources | 0.81 | 0.47 | 0.33 | 0.81 | 0.42 | 0.32 | 0.82 | 0.54 | 0.34 |
| Staff | 0.82 | 0.43 | 0.33 | 0.81 | 0.41 | 0.32 | 0.83 | 0.44 | 0.36 |
| Space | 0.82 | 0.43 | 0.33 | 0.80 | 0.42 | 0.31 | 0.84 | 0.43 | 0.36 |
| Time | 0.81 | 0.54 | 0.32 | 0.78 | 0.60 | 0.29 | 0.83 | 0.47 | 0.35 |

α: Chrobach’s alpha**,** ACT: Alberta Context Tool, ^§^includes those where profession was missing or recorded as “other”

Acceptable values according to the original ACT validation: ^#^α acceptable internal consistency >0.7, *Acceptable item-rest correlations >0.3, ^Acceptable Inter-item correlation values >0.15
