# Supplementary material for: Hospital organizational context and delivery of evidence-based stroke care: a cross-sectional study
Source: Implement Sci. 2019 Jan 18;14:6. doi: 10.1186/s13012-018-0849-z (PMC6339367; doi:10.1186/s13012-018-0849-z)
Supplement: Supplementary file 4 — Table S3. Qualitative results: perceived barriers and facilitators to delivering acute stroke care. (DOCX 14 kb) [file 13012_2018_849_MOESM4_ESM.docx]

**Supplemental Table 3. Qualitative results: Perceived Barriers and Facilitators to delivering acute stroke care**

| **Enablers** | **Barriers** |
| --- | --- |
| **Low context** |  |
| - Committed and enthusiastic multidisciplinary team - Clear pathways, protocols and processes - Support from executive and/or general medicine | - Lack of staff and lack of stable staff - Lack of staff with stroke specific training - Allied health were not specific to the stroke unit and so less engaged - Lack of a stroke care coordinator or medical lead - Lack of education and use of audit and feedback |
| **High context** |  |
| - Committed multidisciplinary team | - Lack of support from other units for care such as thrombolysis |
| - Good communication between team members across disciplines | - Lack of executive support |
| - Strong leadership from either a medical lead, Clinical Nurse Coordinator or both. | - Pressure from management to discharge patients |
| - Commitment to training and education | - Lack of staffing and time |
| - A culture of striving for improvement |  |
| - Use of performance monitoring including audit and feedback |  |
