# Supplementary material for: Hospital organizational context and delivery of evidence-based stroke care: a cross-sectional study
Source: Implement Sci. 2019 Jan 18;14:6. doi: 10.1186/s13012-018-0849-z (PMC6339367; doi:10.1186/s13012-018-0849-z)
Supplement: Supplementary file 5 — Supplemental acknowledgements. List of project contributors not included in the Acknowledgements section of the main manuscript (PDF 85 kb) [file 13012_2018_849_MOESM5_ESM.pdf]

SUPPLEMENTAL ACKNOWLEDGMENTS

Hospital organizational context and delivery of evidence-based stroke care: a cross-sectional study

Authors: Nadine E Andrew, Sandy Middleton, Rohan Grimley, Craig S Anderson, Geoffrey A Donnan, Natasha A Lannin, Enna Stroil Salama, Brenda Grabsch, Monique F Kilkenny, Janet E Squires, Dominique A Cadilhac for the Stroke123 Investigators*

Corresponding Author:
Professor Dominique Cadilhac
School of Clinical Sciences, Monash University, Monash Health Research Precinct Building, Level 3, Hudson Institute Building, 27-31 Wright Street, Clayton VIC 3168.
P: +61 3 8572 2657; F: +61 3 9594 6258; E: dominique.cadilhac@monash.edu

Supplemental acknowledgments

Co-investigators and other contributors to the Stroke123 project or Australian Stroke Clinical Registry (AuSCR) not listed as authors. Note only data obtained in 2013 from AuSCR were used in this current paper from Queensland hospitals in which clinicians completed the baseline questionnaires.

STROKE123 COLLABORATORS AND CONTRIBUTORS

Chief Investigators: Prof **Amanda G Thrift** (Monash University), A/Prof **Steven G. Faux** (University of New South Wales and St Vincent's Health); Prof **Christopher Levi** (University of New South Wales); A/Prof **Vijaya Sundararajan** (University of Melbourne)

Associate investigators: Ms **Sonia Denisenko** (Victorian government); Professor **Helen Dewey** (Victorian government until 2014); Dr **Erin Godecke** (Western Australia Department of Health clinical representative); Ms **Judy Katzenellenbogen** (Department of Health data-linkage representative); Dr **Erin Lalor** (previously of the National Stroke Foundation); Dr **Andrew Lee** (Department of Health South Australia clinical representative); Mr **Mark Longworth** (New South Wales Agency for Clinical Innovation – Statewide Stroke Services Clinical Network up until 2015).

Others:
Queensland Statewide Stroke Clinical Network: **Greg Cadigan; Cindy Dilworth** (until 2013); **Sarah Kuhle**; **John Wakefield** (Queensland Health).

Stroke Foundation: **Kelvin Hill** (Manager, Guidelines Program); **Chris Price** (until December 2012); **Matthew Page** (Queensland StrokeLink Project Officer until 2013); **Helen Branagan** (Queensland StrokeLink Project Officer 2014- 2015), **Jen Egan** (previously the Queensland National Stroke Foundation State Manager until 2014); **Libby Dunstan** (previously the Queensland National Stroke Foundation State Manager until 2017), **Elizabeth Ritchie** (Queensland Quality Improvement and Evaluation sub-committee).

Monash University project staff: **Tharshanah Thayabaranathan, Tara Purvis, Megan Reyneke, Joosup Kim**.

AUSTRALIAN STROKE CLINICAL REGISTRY: hospital site investigators and registry staff
The following site investigators are acknowledged for their contribution to collecting Queensland hospital data on the patients registered in AuSCR as relevant to this aspect of the Stroke123 project up to the end of 2013:

Andrew Wong, MBBS, PhD (Royal Brisbane and Women's Hospital Queensland [QLD] site investigator); **Noel Saines** MBBS, FRACP (The Wesley Hospital QLD, site investigator); **Richard Geraghty**, MBBS, FRACP (Redcliffe Hospital QLD, site investigator); **Pradeep Bambery**, MD, FRCP(G), FRACP (Bundaberg Hospital QLD, site investigator); **Christopher Staples**, MD (Mater Adults QLD, site investigator); **Amanda Siller**, MBBS, FRACP (Queen Elizabeth II Jubilee Hospital QLD, site investigator); **Richard White**, MD, FRCP, FRACP. (Townsville Hospital QLD, site investigator); **Arman Sabet**, MD, FRACP, BSc (Gold Coast Hospital and Robina Hospital QLD, site investigator); **Eva Salud**, MD, AMC CERT (Gympie Hospital QLD, site investigator); **Martin Dunlop**, MBBS, FACRM (Cairns Base Hospital QLD, site investigator); **Nisal Gange**, MBBS, AMC CERT (Toowoomba Hospital QLD, site investigator); **Paula Easton**, BPhty (Hons) (Mackay Hospital QLD, site investigator); **Graham Hall**, MBBS, FRACP (Princess Alexandra Hospital QLD, site investigator); **Sean Butler**, FIMLS, BM Hons, MRCP(UK), FRACP (Prince Charles Hospital QLD, site investigator); **Karen Hines**, BHIM (Caboolture Hospital QLD, site investigator); **David Douglas**, MBBS, M Admin, FRACGP, FAFRM (RACP) (Ipswich Hospital QLD, site investigator); **Suzana Milosevic**, MD, FRACP, AMC CERT (Logan Hospital QLD, site investigator); **Joel Iedema**, MBBS, FRACP (Redland Hospital QLD, site investigator); **Stephen Read**, MBBS, PhD, FRACP (Royal Brisbane and Women's Hospital QLD, site investigator); **Francis Hishon**, RN (Redland Hospital QLD, site investigator).

AuSCR staff: the Florey Institute of Neuroscience and Mental Health
Robin Armstrong†, Leonid Churilov, Alison Dias, Kelly Drennan, Adele Gibbs, Jen Holland, Joosup Kim (Monash University), Charlotte Krenus, Francis Kung, Karen Moss, Kate Paice, Enna Salama†, Sam Shehata, Renee Stojanovic†, Steven Street†, Emma Tod, Kasey Wallis, Julia Watt.

†During the Stroke123 study worked in the role of Queensland project coordinator and AuSCR coordinator
